# The complete chloroplast genome sequence of an economic plant *Salix wilsonii*

**DOI:** 10.1080/23802359.2019.1668311

**Published:** 2019-10-15

**Authors:** Dongyang Wu, Yupeng Wang, Li Zhang

**Affiliations:** College of Information Science and Technology, Nanjing Forestry University, Nanjing, China

**Keywords:** *Salix wilsonii*, chloroplast genome, rubiaceae, phylogeny

## Abstract

In this study, we report the first complete chloroplast (cp) genome sequence of *Salix wilsonii*. The cp genome is 155,026 bp in length, exhibiting a typical quadripartite structure of a large single copy (LSC) region of 83,917 bp, a small single copy (SSC) region of 16,285 bp and a pair of inverted repeats (IRs) region of 27,412 bp. The overall base composition of the cp genome in asymmetric order is A: 32.09%, C: 17.94%, G: 18.65%, and T: 31.31%, and the AT content is 63.4%, whereas the proportions of AT contents in LSC, SSC, and IR regions are 66.53%, 68.99%, and 58.27%, respectively. Further, phylogenetic analysis of 46 species shows that *S. wilsonii* is evolutionarily closest to *Sailx tetrasperma*, and both of them belong to the genus *Salix*.

*Salix wilsonii* is a member of Salicaceae, which consists of 650 species in the world and is divided into three genera, including *Chosenia*, *Populus*, and *Salix* (Tuo-Ya 1995). The genus *Salix* comprises more than five hundred species and most of them are bush. *Salix wilsonii* has an extensive application value. For example, it can be used as an ornamental landscape tree (such as planting with arbor, especially with colored tree species), providing an unanticipated aesthetic effect; the branches are used for weaving and wood can be used to make utensils.

Chloroplasts genomes are one of the essential organellar genomes, which play a prominent part in the investigation of plant evolution and molecular ecology mechanisms (Wang et al. 2018). Approximately 3011 cp genomes are available in the National Center for Biotechnology Information (NCBI) GenBank Organelle Genome Resources (http://www.ncbi.nlm.nih.gov/genome/browse/), of which only 15 are from the *Salix*. In this paper, we describe the assembly and annotation details of the *S. wilsonii* cp genome (accession no. MK748469), which will give valuable information about the molecular identification, genetic diversity and phylogenetic classification in *Salix*.

The sample materials of healthy and fresh leaves of *S. wilsonii* were collected in Nanjing Forestry University (32°04′41.49″N 118°48′23.45″E). Voucher specimen was deposited in the Key Laboratory of Forest Genetics and Biotechnology, Ministry of Education, Nanjing Forestry University (NL2014SW-001). The whole-genome sequencing was conducted on the Pacbio (Pacific Bioscience, USA) platform and the original reads were a mixture of nuclear, chloroplast and mitochondrial genomic DNA. The process of data analysis was described in brief below. First, quality check and filtering of the sequencing data were performed to obtain clean reads. Then, we corrected the clean reads with Canu (Koren et al. 2017). Finally, we used Falcon (Chin et al. 2016) to assembly and used optical mapping data for the improvement of genome assembly. Generally, the cp genomes of different plants are very conservative, especially in higher plants; and their genome size, structure and gene order are very similar as well. Therefore, based on the sequenced *Salix suchowensis* cp genome sequence (NC_026462.1), the contigs (generated by the assembly of the *S. wilsonii* genome) were aligned with the reference sequence by BLASTN (Camacho et al. 2009), and the cp homologous contigs were screened for downstream analysis.

The complete *S. wilsonii* cp genome was assembled to 155,026 bp in length, with LSC region of 83,917 bp, SSC region of 16,285 bp and two IRs region of 27,412 bp. The overall AT content was 63.4% (LSC, 66.53%; SSC, 68.99%; IRs, 58.27%). Using the online program DOGMA (Wyman et al. 2004), a total of 131 genes were identified in the cp genome, including 85 protein-coding genes, one pseudo gene, 36 tRNAs, and eight rRNAs. The majority of these genes were single copy genes, whereas 19 genes existed as double copies, including eight protein-coding genes (ndhB, rpl2, rpl23, rps12, rps19, rps7, ycf15, and ycf2), seven tRNA genes (trnA-UGC, trnI-CAU, trnI-GAU, trnL-CAA, trnN-GUU, trnR-ACG, and trnV-GAC), and four rRNA genes (rrn4.5, rrn5, rrn16, and rrn23). A neighbour-joining phylogenetic tree was constructed based on 76 protein-coding genes that were extracted from cp genomes of 46 species with MEGA7 (Kumar et al. 2016; Yu et al. 2018). As illustrated in Figure 1, the cp genome of *S. wilsonii* is evolutionarily closest to *Sailx tetrasperma*.

**Figure 1.**
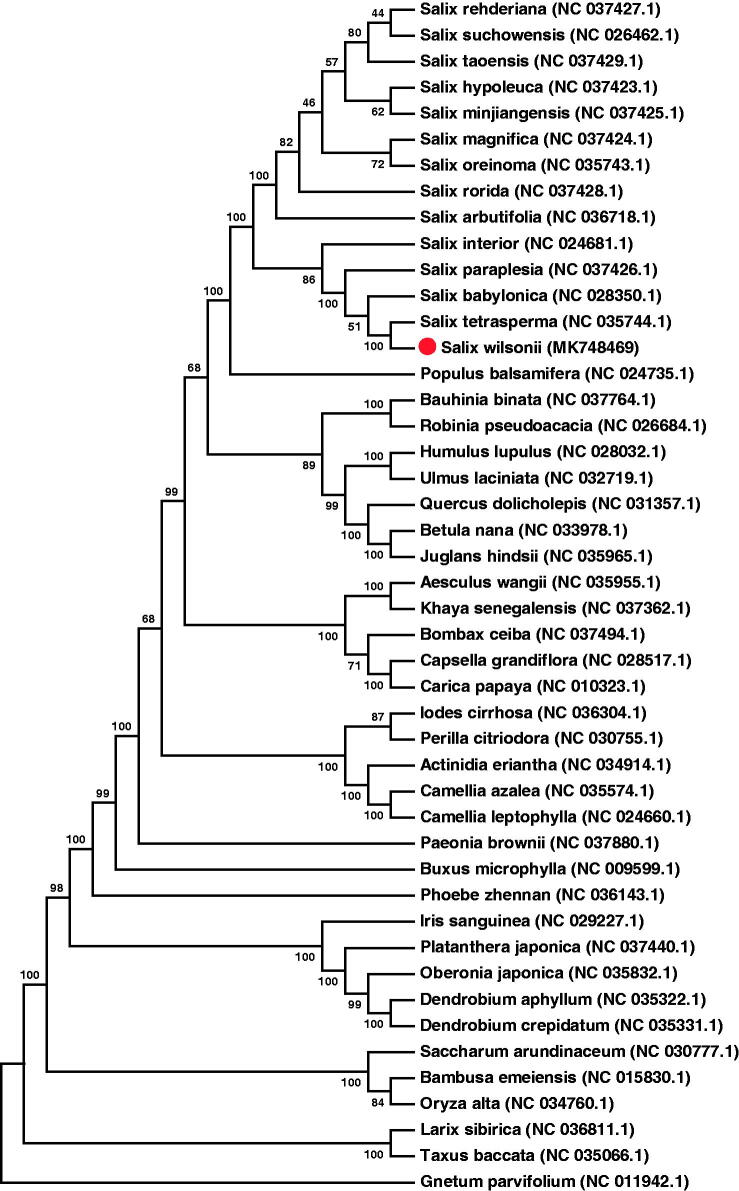
The neighbour-joining phylogenetic tree based on 76 protein-coding genes of 46 species was constructed with MEGA7. The genes were extracted from cp genomes and aligned with Muscle. The bootstrap values from 1000 replicates are listed for each node.

## References

[CIT0001] Camacho C, Coulouris G, Avagyan V, Ma N, Papadopoulos J, Bealer K, Madden TL 2009 Blast+: architecture and applications. BMC Bioinformatics. 10(1):421–420. 20003500 10.1186/1471-2105-10-421 PMC2803857

[CIT0002] Chin C-S, Peluso P, Sedlazeck FJ, Nattestad M, Concepcion GT, Clum A, Dunn C, O'Malley R, Figueroa-Balderas R, Morales-Cruz A, et al. 2016 Phased diploid genome assembly with single-molecule real-time sequencing. Nat Methods. 13(12):1050–1054. 27749838 10.1038/nmeth.4035 PMC5503144

[CIT0003] Koren S, Walenz BP, Berlin K, Miller JR, Bergman NH, Phillippy AM 2017 Canu: scalable and accurate long-re-ad assembly via adaptive, k-mer weighting and repeat separation. Genome Res. 27(5):722. 28298431 10.1101/gr.215087.116 PMC5411767

[CIT0004] Kumar S, Stecher G, Tamura K 2016 Mega7: molecular evolutionary genetics analysis version 7.0 for bigger datasets. Mol Biol Evol. 33(7):1870. 27004904 10.1093/molbev/msw054 PMC8210823

[CIT0005] Tuo-Ya D 1995 Origin, divergence and geographical distribution of salicaceae. Acta Botanica Yunnanica. 17(3):277–290.

[CIT0006] Wang X, Cheng F, Rohlsen D, Bi C, Wang C, Xu Y, Wei S, Ye Q, Yin T, Ye N 2018 Organellar genome assembly methods and comparative analysis of horticultural plants. Hortic Res. 5(1):3. 29423233 10.1038/s41438-017-0002-1 PMC5798811

[CIT0007] Wyman SK, Jansen RK, Boore JL 2004 Automatic annotation of organellar genomes with dogma. Bioinformatics. 20(17):3252–3255. 15180927 10.1093/bioinformatics/bth352

[CIT0008] Yu F, Bi C, Wang X, Qian X, Ye N 2018 The complete mitochondrial genome of Citrus sinensis. Mitochondrial DNA B. 3(2):592–593. 10.1080/23802359.2018.1473738 PMC7799571 33474255

